# Zearalenone toxicosis on reproduction as estrogen receptor selective modulator and alleviation of zearalenone biodegradative agent in pregnant sows

**DOI:** 10.1186/s40104-022-00686-3

**Published:** 2022-04-06

**Authors:** Jianchuan Zhou, Lihong Zhao, Shimeng Huang, Qingxiu Liu, Xiang Ao, Yuanpei Lei, Cheng Ji, Qiugang Ma

**Affiliations:** 1 State Key Laboratory of Animal Nutrition, College of Animal Science and Technology, China Agricultural University, Beijing, 100193 China; 2 Sichuan tieqilishi Food Co., Ltd, Mianyang, 610000 Sichuan province China

**Keywords:** *Bacillus subtilis* ANSB01G, Estrogen receptor, Mycotoxin biodegradation, Pregnant sows, Reproductive performance, ZEA

## Abstract

**Background:**

Zearalenone (ZEA) is a resorcylic acid lactone derivative derived from various *Fusarium* species that are widely found in food and feeds. The molecular structure of ZEA resembles that of the mammalian hormone 17β-oestradiol, thus zearalenone and its metabolites are known to compete with endogenous hormones for estrogen receptors binding sites and to activate transcription of oestrogen-responsive genes. However, the effect of long-term low-dose ZEA exposure on the reproductive response to *Bacillus subtilis* ANSB01G culture for first-parity gilts has not yet been investigated. This study was conducted to investigate the toxic effects of ZEA as an estrogen receptor selective modulator and the alleviating effects of *Bacillus subtilis* ANSB01G cultures as ZEA biodegraders in pregnant sows during their first parity.

**Results:**

A total of 80 first-parity gilts (Yorkshire × Landrace) were randomly assigned to four dietary treatments during gestation: CO (positive control); MO (negative control, 246 μg ZEA/kg diet); COA (CO + *B. subtilis* ANSB01G culture with 2 × 10^9^ CFU/kg diet); MOA (MO + *B. subtilis* ANSB01G culture with 2 × 10^9^ CFU/kg diet). There were 20 replications per treatment with one gilt per replicate. Feeding low-dose ZEA naturally contaminated diets disordered most of reproductive hormones secretion and affected estrogen receptor-α and estrogen receptor-β concentrations in serum and specific organs and led to moderate histopathological changes of gilts, but did not cause significant detrimental effects on reproductive performance. The addition of *Bacillus subtilis* ANSB01G culture to the diet can effectively relieve the competence of ZEA to estrogen receptor and the disturbance of reproductive hormones secretion, and then ameliorate toxicosis of ZEA in gilts.

**Conclusions:**

Collectively, our study investigated the effects of feeding low-dose ZEA on reproduction in pregnant sows during their first parity. Feeding low-dose ZEA could modulate estrogen receptor-α and -β concentrations in specific organs, cause disturbance of reproductive hormones and vulva swelling, and damage organ histopathology and up-regulate apoptosis in sow models. Diet with *Bacillus subtilis* ANSB01G alleviated negative effects of the ZEA on gilts to some extent.

## Introduction

Zearalenone (ZEA), also called F-2 toxin, is a non-steroid estrogen mycotoxin, or a mycoestrogen produced by *Fusarium* species of mold [1]. The structure of ZEA (presence of a phenol ring) resembles that of 17beta-oestradiol (17β-oestradiol) [2]. ZEA and its metabolites, especially alpha- zearalenone (α-ZEL), are well known to compete with endogenous hormones for estrogen receptor binding sites and to activate transcription of oestrogen-responsive genes [3, 4]. The estrogenic activity of α-ZEL is 3–4 times higher than ZEA. Pigs, particularly replacement gilts, are the most susceptible to ZEA [5]. Previous studies have demonstrated that ZEA oestrogenicity cause numerous reproductive dysfunctions in gilts, such as infertility, abortion, ovarian dysfunction, ovulation inhibition, vulvovaginitis, false estrus embryotoxic effects, lesions, ovarian changes, abnormal birth and stillbirth [6–8]. Furthermore, ZEA has also been hepatotoxic, haematotoxic, immunotoxic and genotoxic [9–11].

Growing studies have demonstrated that mycotoxin contamination has been a worldwide problem for food and feed production for a long time [12]. Therefore, the prevention and management of mycotoxins contamination are one of the main goals of the agriculture, food and by-product processing industries [13]. It was well known that mycotoxin biodegradation cites the process of producing non-toxic degradation products by destroying the toxic groups in the molecular structure of the toxin through the action between microbes and their enzymes produced by metabolism and toxins. Several studies demonstrated that some microbes can degrade ZEA [14, 15]. For example, previous studies reported that *Aspergillus* spp. has the ability to transform ZEA over a broad range of ZEA concentrations (5 to 150 μg/mL), and then sulfonation could lead to a less toxic compound [14]. Also, several *Rhodococcus* species are effective in the degradation of economically important mycotoxins, including aflatoxin B1, ZEA, fumonisin B1, T2 toxin and ochratoxin A [15]. Due to its efficient, specific and environmentally protective characteristics and advantages, biodegradation has gained much attention [8, 16, 17]. A strain of *B. subtilis* ANSB01G was screened in our previous work, which could degrade 84.6%, 83.0%, and 66.3% of ZEA in naturally contaminated corn, pig feed, and distiller’s dried grains with soluble, respectively [18, 19]. Some studies had confirmed that *Bacillus subtilis* ANSB01G cultures can ameliorate the effects of ZEA toxicity in sexually-immature or prepubertal gilts [8, 20]. The supplementation of MBA (*Bacillus subtilis* ANSB01G and *Devosia* sp. ANSB714 mixed in ratio) into diets ameliorated these damages induced by dietary toxins (596.86 μg/kg ZEA and 796 μg/kg DON), which MBA administration significantly increased ADG, decreased the vulva sizes, reduced the levels of IgG, IL-8 and PRL in plasma, and regulated apoptosis in ovaries and uteri of gilts [8]. However, the effect of long-term exposure to low-dose ZEA on the reproductive response to the supplementation of *B. subtilis* ANSB01G culture for first-parity gilts is unknown.

Therefore, the objective of this study was to find out the effects of *B. subtilis* ANSB01G culture on reproductive performance, vulva size, serum hormones and organ histopathology in first-parity gilts fed with long-term low-dose corn naturally contaminated with ZEA diets before breeding up to farrowing.

## Materials and methods

### Analysis of dietary mycotoxin

Before the researchers began the experiment, normal and natural moldy corn samples were collected, and then mycotoxins (e.g., ZEA, deoxynivalenol (DON), aflatoxin (AF), and ochratoxin A (OTA)) were detected by the High Performance Liquid Chromatography (HPLC) method as previously described [20]. If the concentrations of ZEA in the moldy corn samples met the requirements, we then purchase the same batch of normal and moldy corn in large quantities. Meanwhile, in the beginning, middle and end of the experiment, corn and feed samples were collected for ZEA, DON, AF and OTA analysis. The HPLC method was used to determine the concentrations of ZEA, DON, AF and OTA in corn and diets according to previous reports [8, 20]. Therefore, in the current study, the detection limits for these mycotoxins were 1.5 μg/kg for the ZEA, 0.1 μg/kg for the AF (aflatoxin B1 (AFB_1_), aflatoxin B2 (AFB_2_), aflatoxin G1 (AFG_1_), and aflatoxin G2 (AFG_2_), 0.02 mg/kg for the DON and 0.5 μg/kg for the OTA, respectively.

### ZEA biodegradation agent

The *B. subtilis* ANSB01G was isolated and confirmed to degrade ZEA efficiently [18, 20]. It was from one batch-fermented at 37 °C for 24 h and then dried at 65 °C. This ZEA biodegradation agent was consisted mainly of 60% carrier (rice husk meal) and 40% *B. subtilis* ANSB01G, using industrial fermentation methods and dry processing techniques. Fermented-dried *B. subtilis* ANSB01G possessed a total viability number of 1 × 10^9^ colony forming unit (CFU)/g.

### Animals, diets and experimental design

A total of 80 first-parity sows (Landrace × Yorkshire) with an average initial weight of 140 ± 6.5 kg were randomly assigned to a factorial arrangement of 2 (normal and moldy diet) × 2 (supplemented with and without ZEA biodegradation agent) treatments: 1) basal control diet containing 60.52% normal corn (CO); 2) basal diet containing 60.52% moldy corn (MO; 164 mg/kg ZEA); 3) CO diet supplemented with 2 g/kg of ZEA biodegradation agent (2 × 10^9^ CFU/kg diet); 4) MO diet supplemented with 2 g/kg of ZEA biodegradation agent (2 × 10^9^ CFU/kg diet). There were 20 replicates of each treatment with one gilt per replicate. Isocaloric and isonitrogenous formulated diets were used in this study. The diets (Table 1) were provided with the nutrients required by the gilts to meet or exceed the requirements of the National Research Council (NRC) [21].

**Table 1:** Diet composition (as-fed basis)

Items	Percentage, %
Ingredients, %	
Corn	60.52
Soybean meal, 43%	16.00
Soybean oil	1.00
Apple pomace	10.50
Soybean hull	8.50
Calcium phosphate	1.70
Limestone	0.94
Lysine (70%)	0.01
Threonine (99%)	0.02
Mineral premix^a^	0.03
Vitamin premix^b^	0.03
Sodium chloride	0.50
Choline (60%)	0.20
Ethoxyquin (66%)	0.05
Total	100.00
Analyzed value	
Crude protein (CP), %	13.02
Metabolic energy (ME), kcal/kg^d^	2700
Standardized ileal digestibility (SID) lys, %^d^	0.57
Lysine (Lys), %	0.66
Methionine (Met) + cysteine (Cys), %	0.42
Threonine (Thr), %	0.51
Tryptophan (Trp), %	0.13
Calcium (Ca), %	0.90
Phosphorus (P), %	0.55

^a^ Provided per kilograms of diet: vitamin A, 17,500 IU; vitamin D_3_, 5000 IU; vitamin E, 40 IU; vitamin K_3_, 5 mg; vitamin B_1_, 5 mg; vitamin B_2_, 12.5 mg; vitamin B_6_, 7.5 mg; vitamin B_12_, 0.05 mg; biotin, 0.2 mg; folic acid, 2 mg; niacin, 30 mg; D-calcium pantothenate, 25 mg;

^b^ Provided per kg diet: Fe, 100 mg as ferrous sulfate; Cu, 17 mg as copper sulfate; Mn, 40 mg as manganese oxide; Zn, 100 mg as zinc oxide; I, 0.25 mg as potassium iodide; and Se, 0.25 mg as sodium selenite

^c^ Mycotoxin biodegradation agent (MBA) was added at the expense of corn

^d^ Calculated values

After the first estrus, all of the gilts were immediately housed in individual stalls (2.0 m × 0.6 m) with the slatted plastic flooring and controlled by environmental control facilities and mechanical ventilation systems. The room was strictly controlled, with disturbances avoided as much as possible, and the inner temperature was kept approximately at 20 to 25 °C by air conditioning system. To facilitate ad libitum access to water and restrict access to diet for gilts throughout the gestation period, a self-feeder and nipple waterer were equipped in each pen. On the d 35 after artificial insemination, pregnancy status was assessed by transabdominal ultrasonography (Honda HS-1600 scanner, Honda Electronics, Tokyo, Japan) in those gilts that had not resumed estrus. After artificial insemination, the gilts were fed treatment diets daily respectively according to the following program: 2 kg/d from d 1 to d 4, 2.4 kg/d from d 5 to d 49, 2.3 kg/d from d 50 to d 90, and 3.2 kg/d from d 90 to d 107 of gestation. The gilts were then transferred to farrowing crates (2.13 m × 0.66 m) and were fed individually from d 108 of gestation until farrowing. From d 108 to d 111 of gestation, 3.5 kg of diet was provided daily. Then the diet was decreased daily by 1.0 kg until farrowing. At parturition, all producing sows were allocated 1 kg of feed and received 1.5 kg of feed on the first postpartum day.

### Experimental methods, sampling, and analysis

On d 60 ± 2 of gestation and after farrowing (114 d), eight sows were randomly selected for each treatment. In this study, blood samples were collected after 12-h fasting for the measurement of serum ZEA and serum hormones. Therefore, on gestation d 60 and d 114, jugular vein blood samples were collected using sterile syringes, and then serum was obtained by centrifugation of blood sample at 3000 r/min for 15 min at 4 °C. In addition, on gestation d 60 ± 2, samples of fecal (at least 0.5 kg) were obtained from each treatment of the same eight sows by rectal massage. All fecal samples were stored at − 20 °C for further analysis of ZEA residues in feces [22]. Residual ZEA and its mentabolites, hormones and estrogen receptor parameters were examined in the serum samples. First, the level of residual ZEA and its metabolites (α-zearalanol, β-zearalanol, α-zearalenol, β-zearalenol and zearalanone) in serum were determined using the HPLC method [22]. Next, serum levels of estradiol (E2), progesterone (PROG), testosterone (T), prolactin (PRL), luteotrophic hormone (LH), and follicle-stimulating hormone (FSH) were measured using the commercial radioimmunoassay kits (Beijing Chemical in Biotech Co., Ltd., Beijing, China) in accordance with the manufacturers’ recommendations. To briefly describe the experimental method, samples with iodine (125I) in monoclonal antibody solution were incubated, and then the binding activity of the incubated samples after aspirating was measured using a gamma counter (GC-1200, USTC Chuangxin Co., Ltd., Zonkia Branch, China). The serum estrogen receptor-α (ERα) and estrogen receptor-β (ERβ) concentrations were determined by a direct solid-phase radioimmunoassay using the commercial kits (BIM Biological Technology Co., Ltd., CA, USA). In addition, the analytical detection limit of the assay was found in the instructions of these kits.

From d 0 and weekly up to d 98 of gestation, from breeding to farrowing, the length, width and height of sows’ vulva were measured and the vulva dimensions were calculated as approximately cylindrical (π × length of vulva × width of vulva × height/4 of vulva) according to the previously described method with slight modifications [20].

At birth, the number and BW of newborn piglets, and the number of stillborn piglets were recorded. After parturition (114 d), 18 sows were randomly selected from CO, MO or MOA treatments (six sows per group), respectively, and all sows were sacrificed using electric shock and bloodletting. The left kidney, liver, spleen, uterus, ovary, and 18 mammary gland samples were collected rapidly by a team of trained personnel. In line with the above procedure, the ERα and ERβ contents in the kidney, liver, spleen, ovary, uterus and mammary glands were determined using the commercial radioimmunoassay kits (BIM Biological Technology Co., Ltd., CA, USA). Then, the samples (ovary, uterus and mammary gland) from each group were fixed in 10% neutral formalin. The tissues were trimmed after fixation and embedded in paraffin. Paraffin samples were cut into 5-μm slices and mounted on the slide, then stained with haematoxylin and eosin (H&E), and examined histopathologically with an Olympus optical microscope.

### Statistical analysis

The variability of the data was expressed using standard error of the mean (SEM). The variability between means was determined using 2 × 2 factorial design analysis. Data were analyzed using the MIXED procedure of SAS 9.1 (SAS Inst., Inc., Cary, NC, USA): fixed factors included toxins content, MBA content, and their interactions, and random factors included experimental period and animal. When interactions were present, means were compared by using Duncan’s range test to determine significant differences among means with a significant level of *P* <  0.05.

## Results

### Mycotoxin content of the experimental diets

ZEA were not detected in the normal corn. The concentrations of ZEA were 467 mg/kg in the moldy corn. The concentrations of ZEA were 10.2 μg/kg, 14.2 μg/kg, 246 μg/kg and 260 μg/kg in CO, COA, MO and MOA diets, respectively. The concentrations of AF were 1.9 and 2.2 μg/kg in MO and MOA, respectively. AF in CO and COA as well as DON and OA in any of the diets were not detected.

### Reproductive performance

Dietary treatments had no effect on the total born piglets per litter, born alive piglets per litter, stillbirth piglets per litter, average initial litter weight or average birth weight (Table 2).

**Table 2:** Effects of MBA on reproductive performance of first-parity gilts when exposed to ZEA^a^

Items	Dietary treatment^b^				Pooled SEM^c^	Source of variation (*P*-value)		
	CO	COA	MO	MOA		Main effect of toxins diets	Main effect of MBA level	Toxins diets × MBA level
Total born, n piglets/litter	16.2	16.25	15.92	16.6	1.08	0.92	0.63	0.57
Born alive, n piglets/litter	14.6	14.67	14.31	15.10	1.16	0.91	0.61	0.55
Stillbirth, n piglets/litter	1.60	1.58	1.62	1.50	0.69	0.99	0.90	0.88
Average initial litter weight, kg	16.55	17.46	16.02	18.29	1.46	0.80	0.13	0.16
Average birth weight, kg	1.04	1.08	1.01	1.12	0.75	0.84	0.18	0.22

^a^ Means represent 20 replications per treatment (*n* = 20/group)

^b^ CO, a positive basal control diet contained 60.52% normal corn; COA, CO + 2 g MBA/kg diet; 3) MO, a negative basal control diet contained 60.52% moldy corn; MOA, MO + 2 g MBA/kg diet; *ZEA* Zearalenone, *DON* Deoxynivalenol, *MBA* Mycotoxin biodegradation agent

^c^ Pooled standard error of the means

### Vulva size

As shown in Table 3, the significant interactions (*P* <  0.05) between toxin and MBA were observed from the vulva size of first gilts at 28, 35, and 98 days of gestation, suggesting the supplementation of MBA effectively ameliorated (*P* <  0.05) the negative effects of ZEA on the reproductive tract. Also, the vulva size in gilts fed with toxin diets was significantly increased (*P* <  0.05) compared with the gilts fed with no toxin diets at 28, 35, and 98 days of gestation. However, dietary treatments had no effect on vulva size in this research.

**Table 3:** Effects of MBA on vulva size of first-parity gilts when exposed to ZEA^c^

Pregnant days	Vulva size, mm^d^				Pooled SEM^e^	*P*-value		
	CO	COA	MO	MOA		Main effect of ZEA	Main effect of MBA	ZEA × MBA
0	11.47	12.26	11.68	12.08	0.714	0.962	0.838	0.473
7	10.10	10.06	11.10	11.88	0.337	0.171	0.305	0.169
14	9.48	9.55	10.08	9.90	0.507	0.841	0.664	0.484
21	9.61	9.33	10.66	9.64	0.408	0.891	0.949	0.649
28	9.68^a^	10.63^ab^	11.59^b^	10.38^ab^	0.412	0.016	0.035	< 0.01
35	9.99^a^	10.69^ab^	11.37^b^	10.70^ab^	0.285	0.063	0.131	< 0.01
42	10.74	10.45	11.37	10.72	0.432	0.603	0.591	0.245
49	10.81	10.49	12.04	11.21	0.492	0.220	0.182	0.065
56	10.55	10.47	11.87	11.02	0.406	0.223	0.317	0.121
63	10.97	10.72	11.47	11.12	0.532	0.721	0.816	0.396
70	11.38	11.12	12.78	11.80	0.656	0.287	0.344	0.135
77	12.59	12.65	13.01	12.71	0.472	0.905	0.973	0.698
84	12.92	12.66	14.35	13.94	0.482	0.159	0.263	0.122
91	16.71	16.84	18.13	16.84	0.855	0.711	0.832	0.482
98	19.59^a^	20.23^ab^	25.76^b^	21.79^ab^	1.316	0.064	0.116	< 0.01

^a,b^ Mean values within a row without a common superscript differ significantly (*P* <  0.05)

^c^ Means represent 20 replications per treatment (*n* = 20/group)

^d^ CO, a positive basal control diet contained 60.52% normal corn; COA, CO + 2 g MBA/kg diet; 3) MO, a negative basal control diet contained 60.52% moldy corn; MOA, MO + 2 g MBA/kg diet; *ZEA* Zearalenone, *MBA* Mycotoxin biodegradation agent

^e^ Pooled standard error of the means

### Serum hormones

There were interactions (*P* <  0.05) in serum concentrations of estradiol (E2) and luteotrophic hormone (LH) between four diets group in the middle of gestation (Table 4). The content of serum E2 in the MO group was significantly increased (*P* <  0.05) than that in other groups. The toxins diets exhibited a significant increase (*P* <  0.05) in serum LH concentration. The addition of MBA to the diet had no effect on serum hormones. No difference was observed in the levels of progesterone (PROG), prolactin (PRL), testosterone (T), or follicle-stimulating hormone (FSH) among all groups.

**Table 4:** Effects of MBA on serum hormones of first-parity gilts when exposed to ZEA^c^

Items		Dietary treatment^d^				Pooled SEM^e^	Source of variation (*P*-value)		
		CO	COA	MO	MOA		Main effect of ZEA	Main effect of MBA	ZEA × MBA
Serum hormones (60 d)	E2, pg/mL	115^b^	117^b^	162^a^	111^b^	14.58	0.1	0.07	< 0.01
	PROG, ng/mL	2.40	2.31	2.47	2.23	0.20	0.82	0.25	0.44
	T, pg/mL	67.2	67.2	62.4	69.3	4.29	0.69	0.34	0.31
	FSH, mIU/mL	5.00	5.07	5.73	4.99	0.42	0.20	0.22	0.10
	LH, mIU/mL	2.94^c^	2.99^c^	3.67^a^	3.34^b^	0.14	< 0.01	0.13	< 0.01
	PRL, mIU/mL	207	204	224	204	11.35	0.59	0.37	0.28
Serum hormones (114 d)	E2, pg/mL	31.03^a^	38.41^a^	76.63^b^	59.35^c^	4.27	< 0.01	0.11	< 0.01
	PROG, ng/mL	3.78^a^	4.11^a^	7.26^b^	5.91^c^	0.13	< 0.01	0.23	< 0.01
	T, pg/mL	68.43	67.44	64.76	66.44	11.90	0.97	0.84	0.90
	FSH, mIU/mL	5.83^a^	6.22^a^	10.82^b^	8.81^c^	0.47	< 0.01	0.43	< 0.01
	LH, mIU/mL	3.22^a^	3.32^a^	5.62^b^	4.41^c^	0.31	< 0.01	0.15	< 0.01
	PRL, mIU/mL	111.09^a^	116.13^a^	260.16^b^	153.56^a^	18.37	0.42	0.07	< 0.01

^a,b^ Mean values within a row without a common superscript differ significantly (*P* <  0.05)

^c^ Means represent eight replications per treatment (*n* = 8/group)

^d^ CO, a positive basal control diet contained 60.52% normal corn; COA, CO + 2 g MBA/kg diet; 3) MO, a negative basal control diet contained 60.52% moldy corn; MOA, MO + 2 g MBA/kg diet; *ZEA* Zearalenone, *DON* Deoxynivalenol, *MBA* Mycotoxin biodegradation agent, *E2* Estradiol, *PROG* Progesterone, *T* Testosterone, *LH* Luteotrophic hormone, *PRL* Prolactin, *FSH* Follicle-stimulating hormone, *MBA* mycotoxin biodegradation agent

^e^ Pooled standard error of the means

At 60 days of gestation, the significant interactions (*P* <  0.05; Table 4) between toxin and MBA were observed from the concentrations of serum E2 and serum LH of gilts. Also, at 114 days of gestation, the significant interactions (*P* <  0.05) between toxin and MBA were observed from the levels of serum E2, PROG, FSH, LH, and PRL of gilts, suggesting the administration of MBA effectively alleviated the physiological dysfunction caused by ZEN. Of note, the concentrations of serum E2, PROG, FSH, and LH in gilts fed with toxin diets were dramatically increased (*P* <  0.05) compared with the gilts fed with no toxin diets after farrowing (114 d). The addition of MBA to the diet did not affect the serum hormones.

### ERα and ERβ concentrations in serum and organs

In the middle of gestation, there were interactions in serum estrogen receptor-α (ERα) and estrogen receptor-β (ERβ) concentrations between toxins and MBA (*P* <  0.05; Table 5). The toxins diets increased (*P* <  0.05) serum ERα and ERβ concentrations, while the addition of MBA did not affect serum ERα and ERβ concentrations.

**Table 5:** Effects of MBA on ERα and ERβ in serum and organs of first-parity gilts when exposed to ZEA^c^

Items, pg/mg		Dietary treatment^d^				PEM^e^	*P*-value		
		CO	COA	MO	MOA		Main effect of ZEA	Main effect of MBA	ZEA × MBA
Serum (60 d)	ERα	1192^a^	1135^a^	1455^b^	1324^c^	64.41	< 0.01	0.38	< 0.01
	ERβ	2247^a^	2240^b^	3085^b^	2585^c^	100.89	< 0.01	0. 49	< 0.01
Serum (114 d)	ERα	1535^b^	1543^b^	1749^a^	1649^ab^	69.35	< 0.01	0.22	< 0.01
	ERβ	1723^b^	1724^b^	2220^a^	1815^b^	99.75	< 0.01	< 0.01	< 0.01
Liver	ERα	4.42	4.61	4.91	4.47	0.45	0.52	0.69	0.33
	ERβ	4.31	4.28	5.19	4.12	0.79	0.54	0.35	0.24
Spleen	ERα	4.21	4.37	5.35	4.87	0.61	0.16	0.61	0.10
	ERβ	3.91^ab^	4.37^ab^	5.11^a^	3.30^b^	0.78	0.15	0.24	< 0.01
Kidney	ERα	4.55	4.67	5.21	4.97	058	0.47	0.88	0.31
	ERβ	4.32	4.32	5.47	4.55	0.77	0.38	0.48	0.22
Uterus	ERα	14.39	14.80	15.26	14.98	0.60	0.35	0.81	0.21
	ERβ	17.98^b^	19.44^ab^	21.63^a^	19.45^ab^	1.35	0.05	0.72	< 0.01
Ovary	ERα	11.89	12.10	12.87	12.05	0.69	0.38	0.56	0.22
	ERβ	13.82^b^	13.84^b^	17.43^a^	13.55^b^	1.36	0.05	0.09	< 0.01
Mammary gland	ERα	7.99	8.14	8.59	8.25	0.45	0.42	0.77	0.24
	ERβ	7.70^b^	8.51^ab^	10.43^a^	8.19^ab^	1.13	0.06	0.30	< 0.01

^a,b^ Mean values within a row without a common superscript differ significantly (*P* < 0.05)

^c^ Means for serum represent eight replications per treatment (*n* = 8/group) and means for organs represent six replications per treatment (*n* = 6/group)

^d^ CO, a positive basal control diet contained 60.52% normal corn; COA, CO + 2 g MBA/kg diet; 3) MO, a negative basal control diet contained 60.52% moldy corn; MOA, MO + 2 g MBA/kg diet; *ZEA* Zearalenone, *MBA* Mycotoxin biodegradation agent, *ERα* Estrogen receptor-α, *ERβ* Estrogen receptor-β, *MBA* Mycotoxin biodegradation agent

^e^ Pooled standard error of the means

On farrowing, significant interactions between four diets group in ERα and ERβ levels of liver and ERβ levels of spleen, ovary, uterus and mammary gland were observed (*P* <  0.05; Table 5). The toxins diets increased serum ERα and ERβ levels (*P* <  0.05). The addition of MBA reduced (*P* <  0.05) serum ERβ content. MO group increased (*P* <  0.05) ERβ content in the ovary, uterus and mammary gland compared with CO group. No differences were tested in ERα or ERβ levels of liver and kidney among all groups.

### Residual ZEA in serum and feces

The ZEA and its metabolites (α-zearalanol, β-zearalanol, α-zearalenol, β-zearalenol and zearalanone) were not detected in the serum (data not shown). Interestingly, as shown in the previous study, the residual ZEA content in feces of gilts fed ZEA-contaminated diets supplemented with MBA was significantly lower than that of gilts fed ZEA contaminated diets.

### Histopathology in organs

**Fig. 1:**
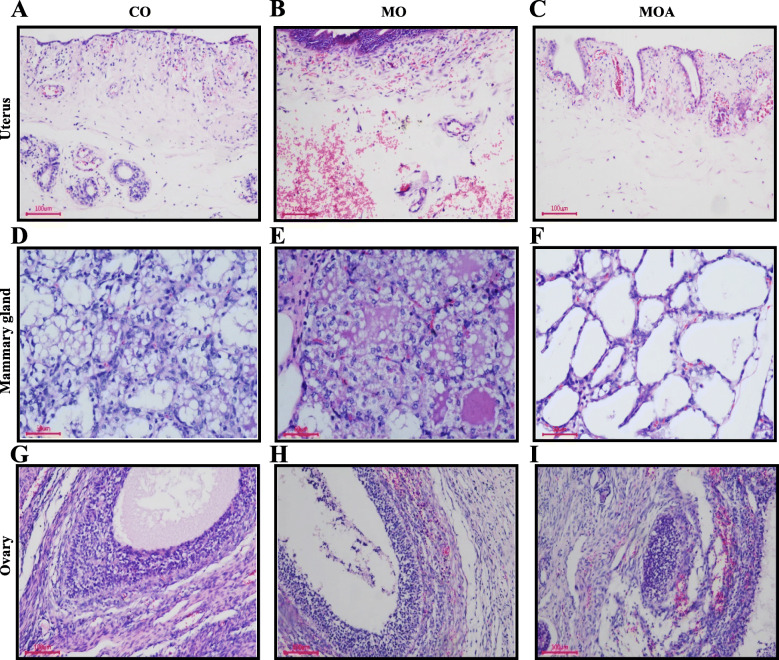
Effects of MBA on histopathology in organs of first-parity gilts when exposed to ZEA. **A** Uterus (CO group): a small amount of inflammatory cell infiltration in inner membrance; capillaries are mildly hyperemic or stagnant. **B** Uterus (MO group): a large amount of inflammatory cell infiltration in inner membrance; severe bleeding in lamina propria. **C** Uterus (MOA group): a small amount of inflammatory cell infiltration in inner membrance; capillaries are mildly hyperemic or stagnant. **D** Mammary gland (CO group): a small amount of reticular secretion in the acinar cavity; glandular epithelial cells swell and become fatty; occasionally, glandular epithelial cells exfoliate and become necrotic. **E** Mammary gland (MO group): a small amount of reticular or mass secretion in the acinar cavity; glandular epithelial cells swell and become fatty; A large number of glandular epithelial cells disintegrate from myoepithelial cell; the glandular cavity contains exfoliated and necrotic glandular epithelial cells. **F** Mammary gland (MOA group): no secretions found in the acinar cavity; glandular epithelial cells swell slightly; a small amount of steatosis occurs. **G** Ovary (CO group): normal structure; a small amount of eosinophils; no obvious histopathological changes observed. **H** Ovary (MO group): bleeding seen in the follicular membrane; a small amount of eosinophils; necrosis in the follicular granulocytes. **I** Ovary (MOA group): bleeding seen in the follicular membrane; a small amount of eosinophils

As shown in Fig. 1, histological structures of all the organs, including the ovary, uterus, and mammary gland, were observed to be normal and essentially lesion-free in the CO group. In contrast, moderate lesions such as swelling, degeneration, inflammatory cell infiltration, hemorrhage, and necrosis were observed in the ovary, uterus and mammary gland to varying degrees in the Mo group. Very interestingly, our results revealed that the administration of MBA significantly ameliorated the lesion of ZEA exposure in the ovary, uterus and mammary gland.

## Discussion

### Reproductive performance

It is generally accepted that pigs are a very sensitive species to ZEA, with prepubertal gilts being the most sensitive stage. Recently, it is well documented that diets with ZEA [7, 23] alone or ZEA with DON together [8, 24–26] might cause detrimental effects on growth performance, cells, tissues and organs, oocyte maturation, antioxidant activities, metabolic process, digestive enzymes, microRNAs, and digestive system in weanling, immature or pre-pubertal gilts. However, fewer studies were conducted to determine the impact of ZEA on the gestation period in first-parity gilts [20, 27–29].

Feeding ZEA contaminated diets (> 1000 μg/kg) decreased the litter size and increased the number of stillborn piglets without any effect on the number of abortion in sows and gits [30]. Yorkshire gilts fed diets containing purified ZEA (3000 μg/kg) after estrus had a higher stillbirth rate, but lower total born piglets or birth weight [31]. Based on the above results, a preliminary conclusion can be made that feeding high ZEA diets increased the stillbirth rate on gilts, but decreased total born piglets, born alive piglets and birth weight. However, we failed to observe the detrimental effects of low-dose ZEA (246 μg/kg) on reproductive performance including stillbirth rate in (Yorkshire × Landrace) first-parity gilts fed naturally contaminated diets from breeding up to farrowing in the present study. Besides, all the gilts were successful in breeding, pregnancy and farrowing. The discrepancy in the stillbirth rate may be due to the different ZEA levels, exposure timing, exposure duration and breed. ZEA levels used in previous studies [27, 29] were 1.2 or 2.8 times higher than those in our study. The very high DON levels (5500 μg/kg or 5700 μg/kg) in previous studies may have a synergistic effect with ZEA. The exposure duration might be nearly 21 d in previous studies, while this was 114 d in the herein study. Besides, it can be seen that the average birth weight among all groups was low due to the large litter size, which even doubled the size compared with previous studies due to the different breed [28]. Based on the findings of European Food Safety Authority (EFSA) Panel on Contaminants in the Food Chain [32], for sexually mature female pigs, a no-observed-effect level (NOAEL) of 40 μg/kg body weight per day was determined based on the results of prolonged cycling and for sows, the highest estimated chronic exposure (P95) for ZEA was 9% of the NOAEL as 3.6 μg/kg body weight per day. This was just consistent with the ZEA level in our study if the assumed average daily feed intake (ADFI) was 3 kg. Therefore, it was supposed that gilts may be more tolerant to low-dose ZEA for a long time than high-dose for short time, especially in the key period during late gestation. Moreover, compared with the CO group, the supplementation of MBA did not affect the reproductive performance of gilts. However, the reproductive performance in the MO group showed a decreasing trend, while the MOA diet group had a higher trend average initial litter weight and average birth weight of gilts than the MO diet.

In our study, according to our description in the Materials and methods section, this MBA reagent contains a large amount of *B. subtilis* ANSB01G culture, which the total viable counts of fermented-dried *B. subtilis* ANSB01G was 1 × 10^9^ CFU/g. Therefore, the supplementation of MBA not only alleviates toxic damage caused by ZEN but also may improve the reproductive performance of gilts. As previously reported, these results have shown that adding *B. subtilis* PB6 to sows’ feed during late gestation and lactation could enhance the growth performance of suckling piglets, and improve the gut health of sows during late gestation [33]. Also, a previous study reported that the administration of *B. subtilis* and *E. faecium* remarkably improved the average daily feed intake of sows and the weight gain of piglets, while significantly decreased the backfat loss and constipation rate of sows [34]. However, during lactation, *B. subtilis* C-3102-fed sows tended to have increased feed intake, but it did not affect sow performance [35], which was consistent with our results.

### Vulva size

Previous research has documented that high concentrations of ZEA (250–5000 μg/kg) can cause vulva swelling in prepubertal gilts [27, 36, 37]. Similarly, several studies also indicated that ZEN resulted in vulvar swelling and vaginal prolapse [20, 38]. In our study, increased vulva size was only observed in the 2^nd^ and 3^rd^ month when gilts fed low dose-ZEA contaminated diets in the current study. However, the absence of ZEN effect in 1^st^ and 4^th^ month were needed to be further studied in addition to the dose. It was well known that female pigs were generally regarded as being a very sensitive species to ZEN. For sexually mature female pigs, a NOAEL of 40 μg/kg body weight per day was identified based on the results of prolonged cycling. Meanwhile, the lowest observed-effect-levels for uterus, ovary and vulva of female pigs ranged from 17 to 200 μg/kg body weight per day and the overall no-observed-effect-level (NOEL) was 10.4 μg/kg body weight per day [32]. Very interestingly, in the present study, the daily feed intake of gilts was 3 kg, while the daily intake of ZEN toxin was 3.5 μg/kg body weight, which was lower than the recommended NOEL value. However, in our study, the significant interactions between toxin and MBA were observed from the vulva size of first gilts at 28, 35, and 98 days of gestation, suggesting the administration of MBA effectively ameliorated the negative effects of low-dose ZEA for a long time on vulva size, which is consistent with previous study [8]. Also, Shi et al. [8] reported that the supplementation of mycotoxin biodegradation agent composed of *Bacillus subtilis* ANSB01G and *Devosia* sp. ANSB714 could effectively relieve the estrogenic swelling in the vulva of gilts caused by the combination of ZEA and DON (596.86 μg/kg and 796 μg/kg, respectively).

### Serum hormones

It is known that ZEA and its metabolites, especially α-ZEL has oestrogenicity to act similarly to the endogenous steroidal sex hormone 17β-oestradiol [39], which may influence the levels of the hormones. Furthermore, ZEA interferes with steroid metabolism mainly by altering the activities of several enzymes [40]. Feeding 2000 μg/kg ZEA diets increased serum PRL level in gilts [41], which was consistent with two previous studies conducted by our lab [8, 20]. Shi et al. [8] and Zhao et al. [20] concluded that serum PRL was significantly increased in gilts fed diets containing ZEA + DON (596.86 μg/kg feed + 796 μg/kg feed; 238.57 μg/kg feed + 0 μg/kg feed, respectively), but no effect on serum FSH, LH or E2. On the contrary, we did not observe any effect of the toxins diets on the levels of serum PRL, E2, PROG, T, or FSH during middle gestation in the present study. However, the toxins diets had a significant effect on serum E2, PROG, FSH and LH after farrowing, which may indicate the time-dependent effect of ZEA on serum hormones. Furthermore, the MO group increased the levels of serum E2 and LH, which was reduced by the addition of MBA in our study. LH and E2 play a vital role in the maturation of follicular cells in the reproductive organs of gilts. LH can induce early follicular membrane cell differentiation, promote follicular dominance formation and induction of ovulation, while E2 can stimulate follicular growth, induce sexual behavior and maintain a normal estrous cycle [42]. The increased serum E2 and LH demonstrated that exogenous ZEA interfered with the normal secretion of reproductive hormones in gilts. Notwithstanding, the results of several studies using purified ZEA in immature gilts were not always consistent. For example, Wu et al. [43] reported that the concentrations of serum LH in immature gilts fed purified ZEA 200 μg/kg, 800 μg/kg, and 1600 μg/kg for 14 d were significantly reduced by 3.61%, 4.01%, and 5.61%, respectively. Fu et al. [44] also revealed that after 14 days of diets contaminated with 1.2 mg/kg ZEA, serum LH level of gilts was decreased compared with the controls. Taken together, these results may indicate that the mature gilts appear to be more sensitive and predisposed to ZEA insult during gestation and farrowing compared to other growth stages.

### ERα and ERβ concentrations in serum and organs

ZEA and its metabolites, especially α-ZEL, compete with the endogenous hormones for estrogen receptor binding sites in vivo and cause serious hyperestrogenism in gilts [39, 40]. Therefore, exposure to ZEA usually leads to precocious puberty and reproductive system disorders [11, 40]. In the herein study, the MO group increased ERα and ERβ levels in serum, ERβ level in the uterus, ovary and mammary gland. No difference in ERα or ERβ concentrations of liver and kidney was observed among all groups. This indicated that uterus and ovary may be the main target organ for ZEA. Although ERα in the uterus was not affected, gilts fed ZEA diets (1500 μg/kg) had 2.0–3.5 fold higher ERβ mRNA and protein abundance in prepubertal gilts [37]. On the contrary, 500–2000 μg/kg ZEA increased ERα levels in uterus and vagina but decreased ERβ levels in the gilts [42]. From the significant changes in ERβ level in ovary, uterus and mammary gland of our results and previous studies, it is possible that ERα may be the primary estrogenic mediator in uterine tissue and ERβ might in part be responsible for uterine growth and maintenance in response to low-dose ZEA, which was mirrored by the lack of reproductive performance difference in this study. The addition of MBA partially relieved these effects on ER.

### Residual ZEA in serum

Previous studies observed that neither ZEA nor its metabolites were detected in liver samples. They explained that low levels (less than 1000 μg/kg) in the diet may be the main reason for the lack of residual ZEA in the serum, liver or muscle tissues of gilts in reserve [8, 20]. The level of ZEA (246 μg/kg) in the present study was even lower than the above studies and the residual ZEA in serum were not detected during middle gestation. This was supported by a previous study, which demonstrated that ZEA may rapidly be excreted by urine with low residue probability. However, several studies observed a very low level of ZEA residue as ng/g in organs and serum. Gilts fed low ZEA diets (200–400 μg/kg BW) had very low residue of ZEA and its metabolites in serum ranged from 4.1 to 18.1 ng/mL after 5.5 h [24]. A previous study confirmed that ZEA was accumulated in the gastrointestinal tissues and liver (1.4–8.6 ng/g) after weeks of exposure in gilts fed low ZEA (1000 μg/kg) diets [2]. A recent study conducted revealed that ZEA accumulated in all intestinal tissues (21.6–153.7 ng/g) in gilts administered ZEA at 40 μg/kg BW [8]. If ZEA mainly accumulated in the gastrointestinal tissues, they may be quickly excreted through urine and feces like the discovery by a previous study [45]. ZEA and its metabolites are excreted mainly as glucuronides via the fecal route. The extensive excretion of bile and the occurrence of a significant enterohepatic circulation are probably the main reason for the relatively long-term persistence of mycotoxin and its derivatives in vivo, especially in pigs [32].

### Histopathology in organs

ZEA may lead to dysfunction of organs and tissues, disrupt homeostasis [25]. Numerous studies indicated that ZEA alone or ZEA with DON together did damage to the histological structures in lamina propria, liver, intestine mucosa and ovaries [4, 26, 40, 46]. In agreement with previous results, moderate lesions such as swelling, degeneration, inflammatory cell infiltration, hemorrhage, and necrosis were observed in the ovary, uterus and mammary gland to varying degrees in the MO group. A review demonstrated that the target organ of ZEA may be the uterus, ovary, then liver and kidney [47]. Similarly, Oliver et al. [48] reported that reproductive tract weight and uterine endometrial gland development were increased in ZEA fed gilts. Several studies observed apparent histological changes in the ovary in gilts fed ZEA diets [49, 50]. Interestingly, compared with the MO pigs, the supplementation of MBA effectively improved multiple organ dysfunction syndromes caused by ZEA [46]. On the contrary, histological examination of the ovaries between the control and high exposure group in gilts fed 290 μg/kg ZEA, did not indicate any differences in the follicle phase distribution [32]. Also, a previous study showed that gilts treated with ZEN diet (40 μg/kg body weight) for six weeks did not alter the architecture of the mucosa or the ratio between goblet and adsorptive cells in the epithelium [4]. Thus, further studies are needed to get insight into this complexity and understand the precise molecular mechanisms underlying the current observations in animal models.

## Conclusions

Combining the above data and discussion, we can conclude that long-term low-dose ZEA did not cause detrimental effects on reproductive performance, but significant interactions between toxin and MBA on serum E2, LH, ERα, ERβ and ERβ of spleen, ovary, uterus and mammary gland in first-parity gilts were found in the present study. These significant interactions suggested that low levels of ZEA in diet adversely affected the above indices, while the addition of MBA (*Bacillus subtilis* ANSB01G) mitigated these damages and the cytotoxicity of the uterus and ovary caused by the dietary toxins. Therefore, *Bacillus subtilis* ANSB01G culture used in this study was considered as a potential application for detoxification of ZEA in animal production.

## Data Availability

The datasets produced and/or analyzed during the current study are available from the corresponding author on reasonable request.

## Abbreviations

AF: Aflatoxin
Ca: Calcium
CFU: Colony forming unit
DON: Deoxynivalenol
DM: Dry matter
E2: Estradiol
ERα: Estrogen receptor-α
ERβ: Estrogen receptor-β
FM: Fumonisin
FSH: Follicle-stimulating hormone
GSH-Px: Glutathione peroxidase
GMA: Glucomannan mycotoxin adsorbent
H&E: Haematoxylin and eosin
LH: Luteotrophic hormone
MBA: Mycotoxin biodegradation agent
MDA: Malondialdehyde
NOEL: No-observed-effect-level
N: Nitrogen
OA: Ochratoxin
P: Phosphorus
PRL: Prolactin
PROG: Progesterone
SEM: Standard error means
SOD: Superoxide dismutase
TUNEL: Transferase-mediated dUTP nick end labeling method
T: Testosterone
ZEA: Zearalenone
